# Experiential avoidance in participants with borderline personality disorder and other personality disorders

**DOI:** 10.1186/s40479-024-00248-1

**Published:** 2024-03-04

**Authors:** Tess C. Gecha, Isabel V. Glass, Frances R. Frankenburg, Carla Sharp, Mary C. Zanarini

**Affiliations:** 1 University of Houston, Houston, TX USA; 2 McLean Hospital, 115 Mill Street, 02478 Belmont, MA USA; 3 Edith Nourse Rogers Veterans Administration Hospital, Bedford, MA USA; 4 Boston University School of Medicine, Boston, MA USA; 5 Harvard Medical School, Boston, MA USA

**Keywords:** Borderline personality disorder, Experiential avoidance, Other personality disorder, Childhood adversity, Adult adversity, Temperament

## Abstract

**Background:**

The present study has descriptive and predictive aims. The descriptive aims were to determine if participants with borderline personality disorder (BPD) reported higher levels of experiential avoidance (EA) than participants with other personality disorders (OPD) as well as determine if non-recovered participants with BPD reported higher levels of EA than participants with BPD who have recovered symptomatically and psychosocially. The predictive aim was to determine if the level of EA reported by participants with BPD was predicted by the severity of aspects of childhood or adult adversity and/or aspects of temperament.

**Methods:**

The Overall Anxiety Severity and Impairment Scale (OASIS) was administered to 248 participants at 24-year follow-up in the McLean Study of Adult Development (MSAD). Adversity and temperament were assessed during index admission using interviews (Revised Childhood Experience Questionnaire [CEQ-R], Adult History Interview [AHI], and the NEO-FFI self-report measure).

**Results:**

Participants with BPD reported significantly higher levels of EA than those with OPD. Within the BPD group, non-recovered participants reported significantly higher levels of EA than recovered participants. Severity of childhood sexual abuse and lower levels of extraversion were found to be significant multivariate predictors of levels of EA in those with BPD.

**Conclusions:**

Taken together, these results suggest that EA is a serious problem for participants with BPD, particularly those who have not recovered. They also suggest that both the severity of childhood adversity and a temperament marked by lower levels of extroversion are significantly related to levels of EA reported by participants with BPD.

## Background

Experiential avoidance (EA) describes the process in which a person is unable to remain in contact with particularly unpleasant internal experiences, such as thoughts, emotions, and bodily sensations, and thus, the person takes measures to avoid or escape these experiences all together [1]. Recent literature has linked EA to a range of psychological disorders, most frequently focusing on anxiety disorders in adults and adolescents [1, 2]. More specifically, studies have shown significant relationships between EA and generalized anxiety disorder and social anxiety disorder [3, 4]. In addition to its associations with anxiety disorders in adults, levels of EA have also been shown to predict anxiety and depressive symptoms in adolescents [5]. EA has also been found to be a partial mediator between behavioral inhibition and social anxiety as well as between the respective effects of social anxiety disorder on quality of life [3, 6].

While there has been extensive research examining EA and anxiety disorders, only five studies have been conducted on the relationship between EA and borderline personality disorder (BPD) or BPD features. This is particularly important as prior studies from our group have found that both avoidant personality disorder [7] and social phobia [8] are common among patients with BPD.

One study looked at the relationship between EA and levels of depression during treatment for BPD in female outpatients (*N* = 81) [9]. Findings showed that EA was positively associated with higher levels of depression before, during, and after treatment, and changes in levels of EA were positively correlated with changes in depression, suggesting that a lower level of EA is related to the reduction of depression during treatment for BPD. Results also confirmed the authors’ hypothesis of directionality, such that while changes in EA predicted changes in depression, there was no such effect for depression on EA.

Another study looked at the relationship between EA and BPD severity to examine whether EA mediates the association between severity of BPD symptoms and non-suicidal self-harm in female inmates (*N* = 117) [10]. Results showed that BPD severity is positively associated with multiple measures of EA, including thought suppression, mental disengagement, behavioral disengagement, denial, and substance use. However, findings did not support the hypothesis that EA mediates the relationship between BPD and self-harm.

A third study looked at the role that EA plays in the relationship between anxiety sensitivity (AS), which is a psychological symptom similar to EA concerning the belief that anxiety will lead to negative somatic, cognitive, and/or social consequences [11], and BPD in a sample of 20 outpatient adults with BPD and 20 outpatient adults without BPD [12]. Findings indicated that EA did mediate the relationship between AS and BPD, and additionally, AS with EA as a mediator accounted for a significant amount of variance in BPD symptoms above and beyond negative affect, affect intensity/reactivity, and impulsivity.

Another study examined EA, emotion dysregulation, and distress tolerance among young adult outpatients (*N* = 40) who met diagnostic criteria for BPD or experienced subthreshold BPD symptoms (i.e., met diagnostic criteria for 3 or 4 symptoms; 13). Partial correlations showed that after accounting for depression, both emotion dysregulation and EA had significant, positive correlations with BPD severity. Moreover, regression analyses revealed that only EA was significantly associated with BPD severity after controlling for depression, demonstrating a specific link between EA and BPD symptomology.

While the previous studies looked at EA in adult populations with BPD, one study looked at EA in adolescents with BPD (*N* = 197) compared to non-BPD inpatient adolescents (*N* = 403) and healthy adolescents (*N* = 92) [14]. Findings revealed that adolescents with BPD had the highest levels of EA, followed by inpatient adolescents with other forms of psychopathology. Moreover, EA was significantly associated with BPD over and above internalizing and externalizing pathology. Of note, two other studies examined EA in adolescents with borderline features, but not with a BPD diagnosis [5, 15]. Findings from both studies showed that the severity of BPD features were associated with higher levels of EA.

Though the aforementioned studies found meaningful relationships between EA and psychological symptoms and behaviors common in BPD, such as depression, anxiety sensitivity, and self-harm, there are still gaps in research looking at the relationship between EA and BPD. To our knowledge, only three studies looked at the specific relationship between EA and a BPD diagnosis [10, 13, 14]. Further, no study has looked at levels of EA in a population of individuals with other personality disorders compared to those with BPD or between subgroups of people with BPD (i.e., those who have met criteria for recovery and those who have not). Lastly, to our knowledge, there have been no studies that have examined experiential or temperamental factors that might confer the risk of developing EA in those with BPD or BPD features, which could have important implications for treatment. To fill these gaps in research, the study first assessed the relationship between severity of anxiety and its impairments among adults with BPD and personality-disorder comparison participants in a large sample of carefully diagnosed participants. It then assessed the relationship between the severity of anxiety and its impairments among adults with BPD who have recovered and those who have not recovered from BPD. Finally, to address the paucity in research examining predictors of EA in those with BPD, the study examined whether certain types of child and adult adversity or temperamental facets predict levels of EA in individuals with BPD.

## Methods

The current study is part of the McLean Study of Adult Development (MSAD), which is a multifaceted longitudinal study of the course of BPD [16]. Participants began enrolling in the study from June 1992 and continued until December 1995. The last follow-up interview was completed in December 2018. The Mclean Hospital Institutional Review Board reviewed and approved study procedures, which have been described in detail elsewhere [16].

Briefly, all individuals were inpatients at McLean Hospital when they began the study. To participate, participants needed to be: (1) between the ages of 18–35; (2) have a known or estimated IQ of 71 or higher; (3) have no history or current symptoms of schizophrenia, schizoaffective disorder, bipolar I disorder, or an organic condition that could cause psychiatric symptoms. Participants with these disorders did not participate in the study because of the difficulty in determining enduring personality patterns in people suffering from psychosis, mania, or a physical illness with serious psychological sequelae.

Participants provided written informed consent after learning about the study procedures. Following consent, a masters-level interviewer blind to the participant’s clinical diagnoses met with each participant for a thorough diagnostic assessment. The assessment included three semi-structured diagnostic interviews: (1) the Structured Clinical Interview for DSM-III-R Axis I Disorders (SCID-I) [17], (2) the Revised Diagnostic Interview for Borderlines (DIB-R) [18], and (3) the Diagnostic Interview for DSM-III-R Personality Disorders [19] (DIPD-R).

There were 12 follow-up waves, each separated by 24 months. At each wave, the same diagnostic battery was administered blind to previous diagnoses and after informed consent was obtained. The follow-up interrater reliability (within one generation of follow-up raters) and follow-up longitudinal reliability (from one generation of raters to the next) of these three measures have been found to be good-excellent [20, 21].

### Revised childhood experience questionnaire

Childhood adversity was assessed using the Revised Childhood Experience Questionnaire (CEQ-R; 18) at baseline. The CEQ-R is a semi-structured interview that assesses the presence and severity of three types of childhood adversity: childhood sexual abuse, other forms of childhood abuse (verbal, emotional, and physical abuse), and seven forms of childhood neglect (physical neglect, emotional withdrawal, inconsistent treatment, denial of participant’s feelings, lack of real relationship with parent or parental figure, caretaker placing subject in parental role, and caretaker’s failure to protect participant). The three types of adversity were scored in the following way: sexual abuse scores range from 0 to 12, other abuse scores range from 0 to 18, and childhood neglect scores range from 0 to 42. Interrater reliability of the instrument has been found to be good-excellent [22].

### Abuse history interview

Adult adversity was measured using the Abuse History Interview (AHI; 23) at baseline and at each follow-up period. The AHI is a semi-structured interview that assesses the presence of adult experiences of violence. During the assessment, the interviewer provides the participant with examples of types of abuse, including emotional (e.g., frequently been shamed or humiliated, repeatedly been frustrated by receiving mixed messages, or often been put in impossible situations), verbal (e.g., often been put down, yelled at, or screamed at), physical (e.g., frequently been slapped around, had things thrown at you, been beaten until the point of bruising), and sexual (e.g., repeatedly been fondled, forced to engage in sexual acts or have intercourse against your will), and asks the participant whether she, he, or they has experienced any of these events. If the participant endorses an experience of abuse, she, he or they are prompted to give a detailed account in her, his, or their own words. Events that were ambiguous were not counted. Interrater reliability of the AHI has been found to be good or excellent [23].

### NEO personality inventory

Temperament was assessed at baseline and at each follow-up period using the NEO Personality Five-Factor Inventory (NEO-FFI; 24). The NEO-FFI is a 60 item self-report measure with well-established psychometric properties that assesses the five-factor model of personality, which includes neuroticism, extraversion, openness to experience, agreeableness, and conscientiousness. Each of the five factors are assessed using 12 items scored on a 5-point Likert scale, ranging from *strongly disagree* (0) to *strongly agree* (4). Total scores for each factor range from 0 to 48. These factors of personality make up the five variables that were used as temperament predictors in our analyses.

### Overall anxiety severity and impairment scale

At the study’s 24-year follow-up, participants completed the Overall Anxiety Severity and Impairment Scale (OASIS; 25), which is a 5-item self-report measure that assesses both the severity of anxiety and the degree to which this level of anxiety interferes with both social and vocational functioning over a one-week time period [25]. The self-report measure uses a 5-point Likert scale ranging from 0 to 4 with higher scores indicating more anxiety and/or impairment. Items are summed to create a total score ranging from 0 to 20.

The OASIS has been found to have good psychometric properties in multiple studies. Two studies using college samples demonstrated that the instrument has good internal consistency, test-retest reliability, and convergent and discriminant validity [25, 26]. A third study using a clinical sample of primary care patients whose physicians referred them to an anxiety disorders treatment study demonstrated in addition to the previous findings that the instrument is unidimensional [27]. With respect to clinical severity, findings from a study using a sample of psychiatric patients with a variety of anxiety disorders support the following cut-off scores: a total score of six indicates moderate anxiety severity, a total score of ten indicates marked severity, and a total score of twelve indicates severe illness [28].

### Definition of recovery

Recovery is defined as a concurrent symptomatic remission of BPD, which we defined as a two-year period in which neither the DIB-R nor the DSM criteria for BPD were met, having at least one emotionally sustaining relationship with a close friend or life partner/spouse, and being able to work or go to school consistently, competently, and on a full-time basis (which included being an unpaid carer in the home or elsewhere) during a two-year follow-up interval.

### Statistical analyses

All analyses were run using Stata version 16.1. Between-group differences of demographic variables were calculated using *t*-tests for continuous variables and Pearson’s chi-squared tests for binary variables. Ordered logistic regression analyses (controlled for sex due to significant between-group differences) and independent samples t-tests were used to assess anxiety severity, frequency, behavioral avoidance, and functional impairment associated with anxiety. Ten bivariate linear regressions were used to examine the predictive qualities of three childhood adversity experiences, two adult adversity experiences, and five temperamental traits on anxiety severity and impairment. All bivariate predictors with a p-level of < 0.05 were then added to a multivariate regression model using backwards deletion to achieve the most parsimonious model. Due to the preliminary nature of this study, statistical significance was set at < 0.01.

## Results

Two hundred and fifty-nine MSAD participants were interviewed at 24-year follow-up. Of these participants, 96% completed the OASIS self-report. Of those participants who completed the OASIS,198 had BPD and 50 had OPD. Table 1 describes demographic results at 24-year follow up for both participants with BPD and participants with OPD. There was a significant between-group difference for sex.

**Table 1:** Demographics of participants with borderline personality disorder and other personality-disordered comparison participants

	BPD (*N* = 198)		OPD (*N* = 50)		BPD vs. OPD	
	***N***	**%**	***N***	**%**	**χ** ^**2**^	***P-value***
Female	164	82.8	32	64.0	8.5398	**0.003**
Non-white	24	12.1	6	12.0	0.0006	0.981
	**Mean**	**SD**	**Mean**	**SD**	**T-test**	***P-value***
Age (years)	50.3	5.7	50.7	7.1	0.3685	0.7128

Table 2 describes the BPD and OPD group comparisons. There were significant differences between participants with BPD (*N* = 198) and those with OPD (*N* = 50) on all the variables studied from the OASIS. More specifically, participants with BPD were more likely to feel anxious, have more severe anxiety, and avoid situations, places, objects, or activities due to anxiety. The anxiety of participants with BPD also interfered significantly more with work, school, home life, social life, and relationships. Total scores on the OASIS were also calculated for both groups, with participants with BPD scoring significantly higher than participants with OPD.

**Table 2:** Mean oasis scores (SD) reported by participants with BPD and other personality-disordered comparison participants

	BPD	OPD	Coefficient	*p*-value	95% Confidence Interval
**How often have you felt anxious?**	2.03 (1.13)	1.22 (0.91)	1.24	< 0.001*	(0.66, 1.82)
**When you have felt anxious, how intense or severe was your anxiety?**	1.71 (1.02)	1.02 (0.87)	1.28	< 0.001*	(0.70, 1.86)
**How often did you avoid situations, place, objects, or activities because of anxiety or fear?**	1.35 (1.17)	0.53 (0.67)	1.39	< 0.001*	(0.78, 2.00)
**How much did your anxiety interfere with your ability to do the things you needed at work, at school, or at home?**	1.27 (1.13)	0.59 (0.88)	1.31	< 0.001*	(0.69, 1.93)
**How much has anxiety interfered with your social life and relationships?**	1.45 (1.19)	0.53 (0.86)	1.66	< 0.001*	(1.02, 2.29)
**Total Score**	7.77 (4.89)	3.86 (3.41)	1.45	< 0.001*	(0.89, 2.01)

Similar patterns emerged between non-recovered and recovered participants with BPD (Table 3). Non-recovered participants (*N* = 119) were significantly more likely than recovered individuals (*N* = 79) to experience anxiety, which was felt to a higher degree, and to avoid situations, places, objects, or activities due to anxiety. Non-recovered participants’ anxiety also caused more functional impairment, and their total OASIS scores were significantly higher than those of recovered participants.

**Table 3:** Mean oasis scores (SD) reported by recovered and non-recovered participants with BPD

	Recovered	Non-recovered	Coefficient	*p*-value	95% Confidence Interval
**How often have you felt anxious?**	1.57 (1.08)	2.33 (1.07)	1.25	< 0.001*	(0.71, 1.78)
**When you have felt anxious, how intense or severe was your anxiety?**	1.21 (0.95)	2.05 (0.93)	1.63	< 0.001*	(1.07, 2.20)
**How often did you avoid situations, place, objects, or activities because of anxiety or fear?**	0.79 (0.91)	1.72 (1.18)	1.53	< 0.001*	(0.98, 2.08)
**How much did your anxiety interfere with your ability to do the things you needed at work, at school, or at home?**	0.68 (0.81)	1.66 (1.15)	1.69	< 0.001*	(1.13, 2.25)
**How much has anxiety interfered with your social life and relationships?**	0.80 (0.95)	1.88 (1.14)	1.84	< 0.001*	(1.27, 2.41)
**Total Score**	5.03 (3.98)	9.59 (4.60)	1.71	< 0.001*	(1.21, 2.28)

Table 4 shows ten bivariate predictors of EA in participants with BPD (three childhood adversity variables, two adulthood adversity variables, and five temperament variables). Four variables were found to be significant bivariate predictors of levels of EA in adults with BPD: severity of childhood sexual abuse, severity of other childhood abuse, severity of childhood neglect, and lower levels of extraversion. When entered into a multivariate model (Table 5), severity of childhood sexual abuse and lower levels of extraversion were found to be significant predictors of levels of EA.

**Table 4:** Bivariate Predictors of Experiential Avoidance in Participants with BPD

	Coefficient	*z*-value or *t*-value	*p*-value	95% Confidence Interval
**Childhood Adversity** Severity of Sexual Abuse	0.54	3.18	0.002*	(0.20, 0.87)
Severity of Other Abuse	0.14	2.22	0.027*	(0.02, 0.27)
Severity of Neglect	0.08	2.27	0.025*	(0.01, 0.14)
**Adult Adversity**				
Rape History	1.34	1.71	0.088	(-0.20, 2.88)
Partner Violence	0.45	0.59	0.555	(-1.04, 1.94)
**Temperament**				
Neuroticism	0.05	1.02	0.310	(-0.05, 0.15)
Extraversion	-0.18	-3.79	< 0.001*	(-0.27, -0.09)
Openness	-0.05	-1.04	0.301	(-0.15, 0.05)
Agreeableness	-0.10	-1.85	0.066	(-0.20, 0.01)
Contentiousness	-0.04	-0.83	0.408	(-0.13, 0.05)

**Table 5:** Multivariate predictors of experiential avoidance in participants with BPD

	Coefficient	*z*-value	*p*-value	95% Confidence Interval
Severity of Sexual Abuse	0.47	2.86	0.005*	(0.15, 0.80)
Extraversion	-0.17	-3.53	0.001*	(-0.26, -0.07)

## Discussion

This study has three main findings. The first is that participants with borderline personality disorder experienced higher levels of experiential avoidance than participants with other personality disorders. Since avoiding social situations is a form of EA, high levels of EA in participants with BPD could, in part, explain why over a 20-year follow-up period, nearly a quarter of participants with BPD were found to be suffering from social isolation (Pucker et al., 2019), which is associated with poor psychiatric and physical outcomes and subjective suffering [29, 30]. Another key piece to EA is that it can lead to significant functional impairment, sometimes leading to job loss, resulting in fewer opportunities for people to build social connections, which have been proven to help mental health outcomes [31–33]. Taken together, these findings illuminate a need to target EA in the treatment of patients with BPD, since reducing levels of EA could lead to fewer difficulties in employment and education.

The second finding is that non-recovered participants with BPD experienced higher levels of EA than recovered participants with BPD. This finding aligns with existing literature, which suggests that non-recovered people with BPD suffer from higher levels of anxiety than those who have recovered [34]. It is unsurprising that non-recovered participants with BPD have higher levels of EA since EA leads to both social and vocational impairment [35], two key components of our definition of recovery: having at least one emotionally sustaining relationship with a close friend or life partner/spouse and being able to work or go to school consistently, competently, and on a full-time basis during a two-year period. Thus, targeting EA in treatment provides a promising avenue for BPD participants who have not yet recovered to build meaningful social connections and succeed vocationally, paving the way toward recovery.

More broadly speaking, and with respect to levels of clinical severity, participants with BPD who have not recovered scored the highest of all groups with a score of 9.59, which is just below the cut-off for severe illness [28]. As a whole, participants with BPD (including both recovered and non-recovered) scored in the moderate to marked severity range (7.77), and the scores of participants with OPD did not even reach moderate severity (3.86).

The third finding of this study is that the severity of childhood sexual abuse and lower levels of temperamental extroversion are associated with higher levels of EA in participants with BPD, suggesting that there exists both a trauma and temperamental influence on EA. However, it is unclear how childhood sexual abuse and extroversion interact with each other to result in an increase of EA in this population. One possible explanation is that less extroverted children may be more vulnerable to abuse experiences as they may be less social and more isolated and are thus possibly less likely to receive support from others, making them “easier victims.” Another explanation could be that after being sexually abused, survivors begin to shy away and/or isolate from people out of fear of being abused again. Regardless of the directionality of influence, and considering these findings, special attention should be paid to individuals with BPD who have reported both childhood sexual abuse and a temperament marked by low levels of extroversion as individuals meeting these criteria appear to be at greater risk of developing EA.

Interestingly, a history of rape or partner violence in adulthood were not found to predict higher levels of EA in participants with BPD. This suggests that there is something about experiencing abuse during childhood that makes it more psychologically harmful in terms of EA than experiencing abuse during adulthood. It could be that those who experienced childhood adversity were abused or not protected by their parents, on whom they were still naturally very dependent, deepening the psychological harm from the maltreatment that would go on to affect levels of EA in the future.

These findings reflect a significant relationship between anxiety-related impairment and BPD, especially among participants with BPD who have not recovered. By identifying this relationship, we have shown that EA may be a useful point of therapeutic intervention for individuals with BPD, especially for those who have not yet recovered. Another important consideration to make when determining who needs additional support is levels of social isolation. In another paper from this sample, we found that borderline patients were significantly more likely over time to be socially isolated than patients with OPD. This was defined as having no emotionally sustaining relationships [36].

More specifically, some of our patients avoided relationships of all kinds, including romantic and/or sexual relationships. In this way, they avoided the desperate and debilitating relationships with romantic partners so common among patients with BPD. However, this avoidance, while self-protective, also led to a lonely life of social and often vocational isolation.

Perhaps the most common psychiatric treatment that targets EA is acceptance and commitment therapy (ACT), which aims to decrease emotional avoidance and increase the capacity for behavior change [37]. It is commonly used for the treatment of people suffering from a variety of disorders that are high in EA, such as panic disorder and other anxiety disorders [37]. Considering ACT’s focus on decreasing avoidance, it could be beneficial to utilize ACT when treating individuals with BPD or incorporate aspects of ACT that target EA into existing evidence-based treatments for BPD, such as dialectical behavioral therapy (DBT) [38] or mentalization-based treatment (MBT) [39].

The current study has several limitations. The first limitation is that all MSAD participants began the study when they were inpatients. The second limitation is that a high percentage of MSAD participants received treatment, typically intermittent treatment as usual in the community, during the 24-year follow-up period [40]. Therefore, it is unclear whether our results would generalize to BPD individuals who are less disturbed or who were not in treatment.

In terms of future research, there is a need for longitudinal research to track the course of anxiety, EA, and its associated functional impairment in BPD and OPD individuals from adolescence into adulthood to gain a better understanding of how pervasive these psychological phenomena are in these populations.

## Conclusions

In sum, these results suggest that anxiety and EA are particularly high in people with BPD, particularly those who have not recovered. They also show that this anxiety and EA interferes with functioning in a variety of realms, making it difficult for some people with BPD to flourish socially and vocationally. Finally, findings suggest that the interplay of severity of childhood sexual abuse and low levels of extroversion is significantly related to levels of EA reported by participants with BPD.

## Data Availability

Requests for data must be made to Dr. Zanarini. Data is held in a secure count at Mass General Brigham, McLean’s parent company.

## Abbreviations

BPD: Borderline personality disorder
OPD: Other personality disorders
EA: Experiential avoidance
